# Pediatric vs. adult stroke: comparative study in a tertiary referral hospital, Cairo, Egypt

**DOI:** 10.1186/s41983-022-00514-5

**Published:** 2022-07-07

**Authors:** Ramy Alloush, Nahed Salah Eldin, Hala El-Khawas, Rania Shatla, Maha Nada, Maha Z. Mohammed, Adel Alloush

**Affiliations:** 1 Department of Neurology and Psychiatry, Faculty of Medicine, Ain Shams University, Cairo, Egypt; 2 Department of Pediatrics, Faculty of Medicine, Ain Shams University, Cairo, Egypt; 3 Department of Geriatrics and Gerontology, Faculty of Medicine, Ain Shams University, Cairo, Egypt

**Keywords:** Pediatric ischemic stroke, Adult ischemic stroke, TOAST criteria, Oxfordshire Community Stroke Project (OCSP), NIHSS, MRI, MRA, TCD

## Abstract

**Background:**

Even though stroke is rare in children, it is associated with serious or life-threatening consequences. Despite its rarity, the occurrence of stroke in children has age-related differences in risk factors, etiopathogenesis, and clinical presentations. Unlike adults, who have arteriosclerosis as the major cause of stroke, risk factors for pediatric strokes are multiple, including cardiac disorders, infection, prothrombotic disorders, moyamoya disease, moyamoya syndrome, and others. The goal of the current study was to compare the characteristics, clinical features, etiology, subtypes, and workup of pediatric and adult strokes.

**Methods:**

This was a hospital-based observational study conducted on 222 participants. All patients underwent a full clinical and neurological examination, full laboratory study, cardiac evaluation, and neuroimaging; CT scan, MRI, MRA, MRV, carotid duplex, and transcranial Doppler (TCD). Ischemic stroke (IS) etiology was classified according to the Trial of Org 10172 in Acute Stroke Treatment (TOAST) criteria, the "proposed classification for subtypes of arterial ischemic stroke in children," and the Oxfordshire Community Stroke Project (OCSP). Stroke severity was determined by the National Institutes of Health Stroke Scale (NIHSS) and PedNIHSS on admission.

**Results:**

The proportion of pediatric ischemic strokes in the current study was 63.4 percent, while hemorrhagic strokes were 36.5%. The majority of the adult patients had ischemic strokes (84.1%), while hemorrhagic strokes were noted in 15.8% of the patients. According to the original TOAST classification, in the current study, the etiology of pediatric IS was other determined causes in 63.6%, undetermined etiology in 27.2%, and cardioembolic in 9.0%. For the adult group, the major stroke subtypes were large artery disease, small vessel disease, cardioembolic, other determined causes, and undetermined etiology at 49.6%, 28.6%, 6.9%, 0.6%, and 12.5%, respectively.

**Conclusions:**

There is a greater etiological role for non-atherosclerotic arteriopathies, coagulopathies, and hematological disorders in pediatric stroke, while adults have more atherothrombotic causes. The co-existence of multiple risk factors in pediatric ischemic stroke is noticed. Thrombophilia evaluation is helpful in every case of childhood stroke. Children who have had a stroke should undergo vascular imaging as soon as possible. Imaging modalities include TCD and Doppler ultrasound, CT, MRI, MRA, and MRV, and cerebral angiography.

## Introduction

Stroke is the second leading cause of death worldwide, accounting for around 5.5 million fatalities per year. Stroke has a high mortality rate, but it also has a high morbidity rate, resulting in up to 50% of survivors being permanently impaired. Thus, stroke is a major public health problem with substantial economic and social ramifications. The public health burden of stroke is expected to rise in the coming decades as a result of population demographic shifts, particularly in developing countries [1]. Although stroke can occur at any age or stage in life, pediatric stroke is a rare condition. However, the high rate of morbidity and mortality in pediatric stroke is ascribed to significant delays in diagnosis, and this is because of a lack of awareness as well as its rarity. Pediatric strokes are exceedingly rare, with an incidence of 1.2 to 2.7 per 100,000 children aged 1 month to 18 years in developed countries [2]. However, it is a major source of persistent neurologic disabilities, such as hemiparesis, epilepsy, and cognitive dysfunction [3]. The proportion of patients with long-term neurologic deficit, and the impact on quality of life and the health care system could be significantly higher in children [4].

The clinical presentation of pediatric stroke is extremely various, depending on age, cause, affected vascular territory, and stroke subtype. Although, focal neurologic deficits are common presentation of ischemic stroke (IS) in children, diffuse manifestations such as seizures, headache, and altered mental status are also possible [5]. The diagnosis of stroke is particularly perplexing in children because of the high prevalence of stroke mimics (migraine, Todd’s paralysis, and psychogenic causes) as history and physical examination alone cannot reliably distinguish stroke and mimics [6]. However, seizures within the acute setting of childhood stroke are common and undiagnosed [7].

Children have a more diverse and larger number of etiological factors and risk factors for stroke that differ significantly from adults [8]. For childhood IS predisposing factors include vasculopathies, such as transient cerebral arteriopathy, arterial dissection, fibromuscular dysplasia, and Moya–Moya Disease [9]. In addition**,** prothrombotic disorders such as protein C and protein S deficiency, antithrombin III deficiency, factor V Leiden mutation. The acquired risk factors for pediatric thromboembolic stroke include iatrogenic causes, acute diseases (sepsis and dehydration), chronic diseases (malignancy, renal, cardiac, collagen and rheumatic diseases) and substance abuse, especially in teenage patients [10]. In addition, stroke subtypes, risk of recurrence, and choices of management differ between both groups. Almost 55% of pediatric strokes are ischemic vs. 80% in adults. In addition, although 20% of adult strokes are hemorrhagic, this stroke type accounts for approximately half of all childhood strokes [11].

With the onset of impairment during childhood and the effect on quality of life for the child and family, the physical and emotional costs are augmented [6]. Affected patient have time urgent, critical needs that present unique challenges to the neurologist, pediatrician, anesthetist, and interventional radiologists. Successful treatment requires rapid recognition and evaluation, imaging, and treatment [12].

This study was designed to determine the stroke subtypes, clinical presentation and the main pathophysiological mechanisms that lead to stroke in children in comparison to adults in tertiary referring hospital for better understanding of underlying mechanisms of stroke so as to improve the prospects for primary and secondary prevention in such cases.

## Methods

This was a hospital-based observational study conducted on 222 participants. The patients were recruited consecutively from the stroke units of Ain Shams University Hospitals and Pediatrics department, Faculty of Medicine Ain Shams University during the period from July 2020 till to January 2022. This study was approved by the ethical committee of Ain Shams University and an informed consent was obtained from all participants and or their caregivers in the study. The diagnosis was made based on the clinical features in combination with brain imaging [13]. The inclusion criteria were patients admitted with the diagnosis of acute stroke within 48 h of onset of the symptoms. Group (A) included pediatric stroke patients whose ages ranged from 1 month to 18 years, and Group (B) included adult stroke patients whose ages above 18 years. Exclusion criteria were patients with perinatal stroke, patients who developed stroke in association with central nervous system infection, patients with traumatic cerebral hemorrhage, and patients with brain tumor, or other central nervous system diseases.

A full history was taken from all patients or their caregivers, the following variables were collected demographic data, vascular risk factors (known or discovered during hospitalization, such as smoking, hypertension, diabetes mellitus, dyslipidemia, obesity, atrial fibrillation [AF] and heart disease), drug intake, presence of patent foramen oval, usage of oral contraceptives, bleeding disorders (congenital or acquired), and any history of previous similar attacks were reported. Special stress on the onset of stroke, and its clinical presentation. All patients were subjected to full neurological and clinical examination to evaluate their neurological condition and to detect any evidence of systemic illness.

All patients were subjected to the stroke protocol and underwent MRI of the brain including diffusion weighted, T1, T2, Flair and gradient echo T2* weighted, together with an MRA of the brain. Brain CT angiography, and MRV were done in some cases. All imaging studies were evaluated by the same neuroradiologist.

All patients were subjected to an electrocardiogram, transthoracic echocardiography (TTE), and/or Transesophageal echocardiogram (TEE), carotid duplex and Transcranial Doppler (TCD).

Baseline investigations (performed upon admission) included a complete differential blood count, blood glucose, C-reactive protein, erythrocyte sedimentation rate (ESR), prothrombin time, activated thromboplastin time and metabolic screening, including liver function test, blood urea nitrogen, creatinine, serum electrolytes, serum iron level and triglycerides/cholesterol serum levels. For inherited thrombophilic disorders, genetic assay was conducted for factor V Leiden G1691A, factor II G20210A, and methyltetrahydrofolate reductase (MTHFR) C677T tests with homocysteine level. Antithrombin III, protein S, and protein C deficiencies were screened. Additional laboratory tests were considered (in some cases for the final diagnosis). Tests for connective tissue disorders, rheumatoid factor (RF), anti-cyclic citrullinated peptide, antinuclear antibodies, lupus anticoagulant (LA), anti-cardiolipins (ACLs), anti-double-stranded DNA, complement components (C3 and C4) were done in some cases. Cerebrospinal fluid analyses, and microbiologic examination by microscopy, culture, polymerase chain reaction (PCR), and d-dimer tests were done in some cases.

The stroke type was classified according to World Health Organization criteria (cerebral infarction, transient stroke, intracranial hemorrhage and cerebral venous thrombosis) [14].

Ischemic stroke etiology was classified according to the TOAST criteria [15]. Large-artery atherosclerosis (LAA) (embolus/thrombosis); clinical findings, and neuroimaging results consistent with LAA and other etiologies have been excluded; (1) Clinical and brain imaging findings of significant (> 50%) stenosis or occlusion of a major brain artery, presumably due to atherosclerosis. (2) A history of TIAs in the same vascular territory, a history of intermittent claudication, the presence of a carotid bruit or diminished pulses. (3) Cortical or cerebellar lesions and brain stem or subcortical hemispheric infarcts > 1.5 cm in diameter on CT or MRI [16].

Cardio-embolism; if there was, one major cardiac risk factor for embolism with no evidence of other stroke subtypes. Small-vessel occlusion; defined as clinical lacunar syndrome, with infarction less than 1.5 cm. Stroke of other determined etiology, if there was evidence of other stroke risk factor (hypercoagulable states or nonatherosclerotic vasculopathy) with the absence of other stroke subtypes features. Stroke of undetermined etiology; if there were more than one potential cause and no etiology found from the investigations [17]. Focal cerebral arteriopathy is a unilateral focal stenosis with irregularity of the large intracranial arteries of the anterior circulation (distal internal carotid artery and/or its proximal branches) [18]. Moyamoya disease is a unilateral or bilateral progressive cerebral arteriopathy with collaterals formation, with radiologic evidence of stenosis or vessel irregularity of large intracranial arteries (distal internal carotid artery, middle cerebral artery, anterior cerebral artery, posterior cerebral artery) supplying the territory of the infarct, and evidence of an excessive collateral network of vessels distal to the occluded arteries [5].

Stroke subtypes in children were categorized according to the TOAST criteria and the ‘‘proposed classification for subtypes of arterial ischemic stroke in children” [19].

Acute ischemic stroke (AIS) was classified according to the presenting clinical features, and the cerebral territory affected with the Oxfordshire Community Stroke Project (OCSP); Total anterior circulation syndrome (TACS), Partial anterior circulation syndrome (PACS), Posterior circulation syndrome (POCS), and lacunar syndrome (LACS) based on their maximum neurological defects [20]. Stroke severity was determined by National Institutes of Health Stroke Scale (NIHSS) and PedNIHSS on admission [21, 22].

### Statistical analysis

Results were expressed as mean ± standard deviation or number (%). Comparison between categorical data [number (%)] was performed using Chi square test or Fisher exact test instead if cell count was less than five. Test of normality, Kolmogorov–Smirnov test, was performed to measure the distribution of data. Comparison between not normally distributed data (variables) in the two groups was performed using Mann Whitney test. Statistical Package for Social Sciences (SPSS) computer program (version 19 windows) was used for data analysis. p value ≤ 0.05 was considered significant.

## Results

**Fig. 1:**
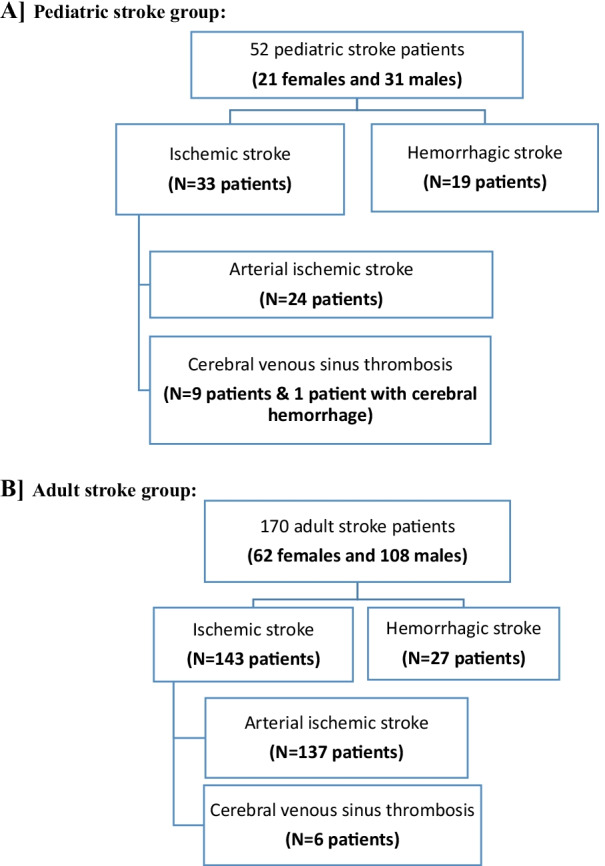
Flowchart of (**A**) pediatric stroke group and (**B**) adult stroke group

This study included two groups: Group (A) pediatric stroke and group (B) adult stroke. Pediatric and adult patient identification and classification are shown in the flowchart in (Fig. 1).

The pediatric group consisted of 52 children, 31 males and 21 females; with median age of 6 years and 3 months; age range from 2 months to 18 years. Other demographic data for this together with risk factors and significant medical history are stated in (Table 1).

**Table 1:** Demographic, vascular risk factors, and comparisons between pediatric ischemic and hemorrhagic strokes

Variables	Total cohort 52 Patients	Ischemic stroke 33 Patients	Hemorrhagic stroke 19 Patients	*p* value
Age median age, [age range]	6 years 3 months (2 months–18 years)	16 years (2 months–18 years)	7 years (3 months–18 years)	0.207
Sex				
Boys Girls	31 (59.6%) 21 (40.3%)	16 (48.5%) 17 (51.5%)	15 (78.9%) 4 (21.1%)	
Hypertension	1 (1.9%)	1 (3.0%)	0 (0.0%)	0.444
Cardiac diseases				
Patent foramen ovale Atrial septum defect Ventricular septal defect Ebstein anomaly^a^	5 (9.6%) 1 3 1 2	1 (3.0%) 3 (9.1%) 1 (3.0%) 2 (6.1%)	0 (0.0%) 0 (0.0%) 0 (0.0%) 0 (0.0%)	0.444 0.176 0.444 0.274
History of previous stroke	4 (7.6%)	3 (9.1%)	1 (5.3%)	0.618
History of DVT	1 (1.9%)	1 (3.0%)	0 (0.0%)	0.444
Vasculitis	2 (3.8%)	2 (6.1%)	0 (0.0%)	0.274
Infection^b^	6 (11.5%)	5 (15.2%)	1 (5.3%)	0.282
Drug abuse	1 (1.9%)	0 (0.0%)	1 (5.3%)	0.183

By Mann Whitney test and Chi square test

^a^This boy had two cerebral strokes within four months; the first one was an arterial ischemic attack and the last one was cerebral venous thrombosis

^b^COVID-19 infection in three patients, Herpes encephalitis in one patient, acute mastoiditis in one patient, and tuberculous meningitis with ventriculitis in another

The adult group consisted of 170 patients, 108 were males (36.5%) and 62 were females (63.5%). The median age at the time of presentation was 64 years and 6 months, and the age range was 26–92 years. Other demographic data for this group together with risk factors and significant medical history are stated in (Table 2).

**Table 2:** Demographic and vascular risk factors of both types of stroke in an adult group

Variables	Total cohort	Ischemic stroke	Hemorrhagic stroke	p
Median age, [age range]	64 years 6 months (26–92)	56 years (26–92 )	43 years (40–85)	0.942
Sex	170	143 (84.1%)	27 (15.9%)	
Male Female	108 (63.5%) 62 (36.4%)	91 (63.6%) 52 (36.4%)	17 (63.0%) 10 (37.0%)	
Hypertension	116 (68.2%)	100 (69.9%)	16 (59.3%)	0.275
Diabetes	85 (50%)	75 (52.4%)	10 (37.0%)	0.142
Dyslipidemia	77 (45.2%)	71 (49.7%)	6 (22.2%)	0.009*
Cardiac diseases				
Arrhythmia (AF) Hypokinesia Dilated LA Valvular lesions	55 (32.3%) 20 21 15 4 2	51 (35.7%) 20 (14.0%) 21 (14.7%) 15 (10.5%) 4 (2.8%) 2 (1.4%)	4 (14.8%) 0 (0.0%) 0 (0.0%) 0 (0.0%) 0 (0.0%) 0 (0.0%)	0.034* 0.039* 0.028* 0.078 0.379 0.537
Intracardiac masses				
Smoking habits	25 (14.7%)	22 (15.4%)	3 (11.1%)	0.565
History of previous stroke	43 (25.2%)	38 (26.6%)	5 (18.5%)	0.377

By Mann Whitney test and Chi square test, *p value was significant

In comparison of the clinical presentations of IS in both pediatric and adult groups, diffuse manifestations, especially headache, vomiting, seizures, altered consciousness, and fever, were significantly common in pediatric IS patients, while focal manifestations, aphasia, dysarthria, neuro-ophthalmologic signs, facial palsy, hemiparesis, hemianesthesia, and ataxia/gait abnormality, were significantly higher in adult IS patients. Stroke severity in both pediatric and adult IS was assessed with the PedNIHSS and NIHSS scores and was similar (Table 3).

**Table 3:** Clinical presentations of ischemic stroke in both groups

Clinical presentations	Pediatric 33 Patients	Adult 143 Patients	p value
Diffuse manifestations			
Headache	13 (39.4%)	6 (4.2%)	0.001*
Vomiting	11 (33.3%)	10 (7.0%)	0.001*
Seizure	12 (36.4%)	0 (0.0%)	0.001*
Fever	4 (12.1%)	0 (0.0%)	0.001*
Altered conscious level	10 (30.3%)	23 (16.1%)	0.059
Dizziness	4 (12.1%)	13 (9.1%)	0.595
Focal manifestations			
Aphasia	3 (9.1%)	31 (21.7%)	0.099
Dysarthria	1 (3.0%)	82 (57.3%)	0.001*
Neurologic–ophthalmologic signs^a^	0 (0.0%)	30 (21.0%)	0.004
Facial palsy	8 (24.2%)	90 (62.9%)	0.001*
Ataxia/gait abnormality	1 (3.0%)	30 (21.0%)	0.015*
Hemiparesis	21 (63.6%)	116 (81.1%)	0.029*
Hemianesthesia	8 (24.2%)	79 (55.2%)	0.001*
NIHSS, PedNIHSS			
Mild (1–4)	12 (36.3%)	55 (38.5%)	0.001*
Moderate (5–15)	16 (48.4%)	77 (53.8%)	
Moderate to severe (16–20)	1 (3.0%)	11 (7.7%)	
Severe (21–42)	0 (0.0%)	0 (0.0%)	

By Chi square test and Fisher exact test

^a^Neurologic–ophthalmologic signs (squint, papilledema, visual field defect), *p value was significant

Children with HS had more significant diffuse clinical manifestations, such as headaches, vomiting, and seizures. While the focal manifestations, especially dysarthria, facial palsy, hemiparesis, and hemianesthesia, were significantly common in adult HS patients. Stroke severity in both pediatric and adult HS was assessed with the PedNIHSS and NIHSS scores in the adult HS group with a low score in the pediatric group (Table 4).

**Table 4:** Clinical presentations of hemorrhagic stroke in both group

Clinical presentations	Pediatric group 19 Patients	Adult group 27 Patients	p
Diffuse manifestations			
Headache	13 (68.4%)	13 (48.1%)	0.172
Vomiting	9 (47.4%)	8 (29.6%)	0.220
Seizure	9 (47.4%)	3 (11.1%)	0.006*
Neck stiffness	4 (21.1%)	4 (14.8%)	0.583
Fever	1 (5.3%)	0 (0.0%)	0.228
Altered conscious level	4 (21.1%)	8 (29.6%)	0.514
Dizziness	6 (31.6%)	6 (22.2%)	0.477
Focal manifestations			
Aphasia	2 (10.5%)	5 (18.5%)	0.457
Dysarthria	1 (5.3%)	13 (48.1%)	0.002*
Neurologic–ophthalmologic signs^a^	4 (21.1%)	2 (7.4%)	0.176
Facial palsy	4 (21.1%)	13 (48.1%)	0.061
Ataxia/ gait abnormality	3 (15.8%)	2 (7.4%)	0.368
Hemiparesis	9 (47.4%)	16 (59.3%)	0.425
Hemianesthesia	7 (36.8%)	14 (51.9%)	0.314
NIHSS, PedNIHSS			
Mild (1–4)	4(21.0%)	8 (29.6%)	0.004*
Moderate (5–15)	8 (42.1%)	15 (55.6%)	
Moderate to severe (16–20)	0 (0.0%)	4 (14.8%)	
Severe (21–42)	0 (0.0%)	0 (0.0%)	

By Chi square test

^a^Neurologic–ophthalmologic signs (squint papilledema, visual field defect), *p value was significant

In this study, hypercoagulable disorders, either genetic or acquired, were rarely seen in adult IS, and although it was a major stroke risk factor in 10 patients (30.3%) of pediatric IS (Table 5).

**Table 5:** Role of thrombophilia in ischemic stroke in both groups

Variable	Children 33 Patients	Adult 143 Patients	p
Two or more genetic traits	3 (9.0%)	0	0.001*
Antiphospholipid antibodies	2 (6.0%)	2 (1.3%)	0.105
Protein C deficiency	2 (6.0%)	0	0.003*
Protein S deficiency	2(6.0%)	0	0.003*
Antithrombin deficiency	1 (3%)	0	0.037*
Factor V Leiden	5 (15.1%)	0	0.001*
Factor II G20210A	1 (3%)	0	0.037*
MTHFR gene	7 (21.2%)	0	0.001*
Elevated Homocysteine level	1 (3%)	0	0.037*
B-Fibrinogen gene 455G/A mutation	1 (3%)	0	0.037*

By Chi square test and Fisher exact test, *p value was significant

Large artery atherosclerosis was a common cause of adult stroke in this study. Twenty-one children and one adult were allocated to the “other determined etiology” category. Multiple probable stroke etiologies were identified in 8 children (24.2%) and 18 (12.5%) adults (Table 6).

**Table 6:** Ischemic stroke subtypes and etiology (TOAST) in both groups

Ischemic stroke	Children 33 Patients	Adult 143 Patients	p
Large artery disease	0	71 (49.6%)	0.001*
Cardioembolic	3 (9.0%)	10 (6.9%)	0.118
Small artery disease	0	41 (28.6%)	0.001*
Other determined etiology	21 (63.6%)	1 (0.6%)	0.001*
Multiple cause No identified cause	8 1	18 0	0.035*

By Chi square test and Fisher exact test, *p value was significant

According to the OCSP classification, approximately 37.5% of pediatric IS had PACS, 29.1% had TACS, and 25.0% had POCS, followed by (8.3%) having LACS (Table 7). Similarly, 32.1% of adult IS suffered from TACS, 25.5% from LACS, 23.5% from POCS, and 19.2% from PACS. There was a significant difference between both groups, as PACS was a common clinical subtype in pediatric IS, while LACS was the common subtype in adult IS.

**Table 7:** Clinico-radiological ischemic stroke subtypes in both groups (OCSP classification)

Characteristics	Children (n =24)	Adult (n = 140)	p
Clinical–radiological subtype^a^			
TACS	7 (29.1%)	45 (32.1%)	0.009*
PACS	9 (37.5%)	27(19.2%)	
LACS	2 (8.3%)	35(25.0%)	
POCS	6 (25.0%)	33 (23.5%)	

By Chi square test

^a^No infarcts were present in some patient, *p value was significant

Etiologically, the causes of pediatric cerebral venous sinus thrombosis (CVST) were prothrombotic state (5/10), postinfectious (COVID) (1/10), hematological disease (1/10), vasculitis (1/10), congenital heart disease (1/10), and undefined disease (1/10). In contrast to the adult CVST group, postinfectious (2/6), hypercoagulable state (1/6), contraceptive pills (1/6), and undefined disease (2/6).

For the pediatric HS, the final etiological diagnosis was an AVM in 11 patients (57.8%), a cerebral aneurysm in 5 (26.3%), and a hematological etiology in one case and vasculitis in another case. In adult HS, hypertension (17 cases, 62.9%), cerebral aneurysm (5 cases, 18.5%), and cerebral amyloid angiopathy (2 cases, 7.2%) are the most common causes. Despite comprehensive investigations, undetermined etiology was reported in one case of children and six cases (22.2%) of adults (Table 8).

**Table 8:** Etiology of hemorrhagic stroke in both groups

Cerebral hemorrhage	Children 19 Patients	Adult 27 Patients	p
Hypertension	0	17 (62.9)	0.001*
Cerebral amyloid angiopathy	0	2 (7.2%)	0.225
Vascular malformation	11 (57.8%)	2 (7.2%)	0.001*
Aneurysm	5 (26.3%)	5 (18.5%)	0.528
Hematological disease (HLA)	1 (5.2%)	0	0.228
Vasculitis (JRA)	1 (5.2%)	0	0.228
Cryptogenic	1 (5.2%)	6 (22.2%)	0.115

By Chi square test and Fisher exact test, *p value was significant

## Discussion

The incidence of the two main subtypes of stroke (ischemic and hemorrhagic) is different in adults and children. According to the current study, in childhood, a slight predominance of the IS vs. HS was found at 63.4% and 36.5%, respectively, but in adults, 84.1% had an ischemic stroke and 15.8% had a hemorrhagic stroke. Similar results were reported by two different studies [23, 24], where 58.6% vs. 38.6% or 64% vs. 36% for pediatric stroke. However, in studies reporting strokes in children globally, both types of stroke are equally common [25]. In addition, Chiang and Cheng’s [26] results were closer to global reports; hemorrhagic stroke was 43.6%, ischemic stroke was 36.1%, and TIA was 5%. The differences in these studies are undoubtedly due to a diverse range of researches using different methodologies, study populations, and inclusion criteria. In addition, there is an inherent selection bias, because the bulk of these pediatric stroke researches were conducted at tertiary children's medical centers. However, the current results agreed with the worldwide incidence of adult ischemic stroke, subarachnoid hemorrhage, and intracerebral hemorrhage, 80–85%, 1–7%, and 7–27%, respectively [27, 28]. In addition, approximately 87% of strokes in the United States are ischemic, intracerebral hemorrhage in 10% and subarachnoid hemorrhage in 2% [29].

The male predominance observed in the present study was between boys (59.6%) and girls (40.3%) in the pediatric group, and 63.5% of the patients were males and 36.4% were females in the adult group. Similar findings have been previously reported in the Canadian Pediatric Ischemic Stroke Registry among patients with IS. There was a predominance of males (55%) compared with Canadian children [30]. In addition, Tan and colleagues discovered a male to female ratio of 1.9–1, which was consistent with many previous studies of ischemic stroke in Asia and elsewhere [31]. Hormonal factors may predispose males to early thrombotic stroke and can explain this gender predominance [32].

Children with stroke have remarkable differences in clinical presentation compared with adults; in the current study. Diffuse manifestations, especially headache, vomiting, seizures, altered consciousness, and fever, were significantly common in both subtypes of pediatric stroke, while focal manifestations, aphasia, dysarthria, neuro-ophthalmologic manifestations, facial palsy, hemiparesis, hemianesthesia, and ataxia/gait abnormality, were significantly higher in the adult group. Seizures at stroke onset are more common in children. Many studies have reported that 15–25% of children, particularly those under the age of six, present with seizures [2, 5, 33]. The enhanced excitability of the immature brain might favor seizure propagation in the context of acute stroke. In addition, the immature central nervous system may not demonstrate focal signs, and hemiparesis may not be apparent until a child is older than 6 months of age [34]. The occurrence of some presenting features, such as speech disturbance and headache, might vary with age because of age-related developmental changes. Headache at presentation was associated with older ages as it is largely self-reported relying on the development of adequate communication skills.

The leading risk factor for pediatric IS in the present study was vascular disorders, followed by hematological and prothrombotic disorders. Similarly, according to a study of 156 cases of IS by Jeong et al. (2015), the most common risk factors were arteriopathy in 83 cases, cardiac disease in 23 cases, prothrombotic conditions in 14 cases, and hematologic disease in 6 cases [35]. In addition, during the global COVID-19 pandemic, there were several reported case scenarios of IS occurring in children, as stated in our results, who either suffered acute respiratory distress syndrome caused by SARS-CoV-2 or who presented with IS and other symptoms as part of the pediatric multisystem inflammatory response in children associated with COVID-19 [36]. This might be explained by an uncontrolled inflammatory response and a cytokine storm following infection with SARS-CoV-2 [37]. For the adult group, the greatest risk factor in the current study was hypertension, followed by diabetes mellitus and dyslipidemia. This data agrees with many Egyptian studies, showing similar results as ours [38–40].

It has been widely recognized that stroke etiology differs between children and adults [19]. The main findings of the current study are that the subtypes of ischemic stroke are distinct in children and adults. According to the modified version of the TOAST classification, the first three subtypes (large artery atherosclerosis, cardioembolic, and small vessel disease) accounted for the majority of adult strokes (85.3%). In contrast, only 9% of children were accounted for within these three subtypes. The high prevalence of diffuse atherosclerosis, extracranial/intracranial stenosis and occlusion in this study may explain the high percentage of LAA in Egyptian patients compared to other studies. It is also well recognized that the prevalence of asymptomatic internal carotid artery stenosis (ICAS) was 13.1%, which is relatively high in Egypt [38]. In China, population-based studies using TCD have shown asymptomatic ICAS to be present in about 6–7% of healthy adults [41]. In Western countries, studies have shown asymptomatic ICAS in approximately 9% of the Spanish population [42]. In addition, El-Zayat et al. (2020) found that intracranial significant stenosis is more prevalent than extracranial in 61 acute ischemic stroke Egyptian patients [43]. Lately, Khedr et al. (2021) detected extracranial carotid atherosclerotic changes in 119 patients (64.3%) out of 185 Egyptian patients with acute cerebrovascular stroke [40]. These frequencies were higher than studies population in Pakistan, India and Iran [44–46].

In Egypt, there is a higher prevalence of DM (75.4%) than in Pakistan (37%), Asians (37%), Hispanics (50%), Caucasians (28%), and African Americans (31%). Similarly, there was a slightly higher prevalence rate of dyslipidemia (68.4%) in the Egyptian population, much higher than the same ethnic groups [47, 48]. These findings highlights underscore the importance of early risk factor detection and primary prevention, given the premature onset of cerebrovascular complications.

The majority of children were classified within the “other determined etiology” subtype vs. only one adult patient. Etiology was undetermined in 12.5% of adult cases, compared with 27.2% of children cases. Previous studies comparing stroke etiology in children and adults found more strokes due to undetermined causes in children than in adults, although with differing percentages. Other causes of stroke were detected in 49% of children and 44% of young adults by Williams and colleagues [49], whereas Wraige et al. [19] reported other causes of stroke in up to 80% of children and only 16% of young adults. In addition, Bigi and colleagues [50] found that 52% of children had strokes due to “other determined causes” according to the original TOAST classification, compared to 29% of adults.

In the current study, one or more prothrombotic states have been identified in 48.4% of children presenting with IS and 50% of children with CVST. In agreement with these data, Barnes and Deveber identified prothrombotic abnormalities in 20–50% of children presenting with AIS and 33–99% of children with CVST [51].

Infarcts in children, such as in adults, are most typically found in the anterior circulation. In the current study, according to the OCSP classification, PACS was the most common clinical subtype in pediatric IS (37.5%), while TACS was the most common subtype in adult IS (32.1%). Simonetti and colleagues detected stroke location in 2,768 pediatric AIS cases; 507 (18%) were located in the posterior circulation, 1931 (70%) in the anterior circulation, and 330 (12%) involved both. PCAIS is less common than ACAIS in the pediatric population. Although, children with PCAIS have lower stroke symptom severity at presentation, but they may have an increased stroke recurrence risk, than those with ACAIS [52]. These results highlight the importance of detailed clinical–radiological evaluation; MRI, MRA, MRV, duplex, and TCD of pediatric stroke patients.

Unlike adults who tend to have more cases of primary hypertensive ICH, children tend to suffer from secondary ICH due to vascular malformations or coagulopathy. For the pediatric HS in the current study, the most common etiological diagnosis was an AVM and cerebral aneurysm. However, for adult HS, hypertension was the most common cause, followed by cerebral aneurysm and cerebral amyloid angiopathy. In agreement with these findings, Beslow and colleagues detected that the causes of 22 children were arteriovenous malformation (40%), cavernous angioma (18%), aneurysm (14%), coagulopathy (14%), and others (18%). Acute pediatric hemorrhagic stroke management should include MRA whenever the patient is stable. In addition, unless an alternative cause is identified (as hemophilia), all pediatric patients with hemorrhagic stroke should ultimately have DSA before the hemorrhage is considered idiopathic [53].

Up to 90% of strokes could be avoided if vascular risk factors were controlled [29]. Identifying the cause of the first stroke, whether arterial occlusion or a transient ischemic episode, and developing multidisciplinary techniques to reduce those causes are critical steps in preventing future stroke risk factors. In addition, childhood stroke is well understood to be multifactorial, and atherosclerosis, the most common cause of IS in adults, is extremely rare in children. Thus, a comprehensive diagnostic evaluation is critical for prompt diagnosis and treatment to reduce the risk of brain damage and disability in children following a stroke.

This study had some probable limitations. The number in the pediatric group was relatively small due to the rarity of pediatric stroke. Assessing stroke severity is more difficult in children than in adults, and therefore, comparisons between children and adults might be limited to patients with extremes on the scale. Finally, because TEE was not performed on children, cardioembolic might be underdiagnosed in the pediatric group. Nevertheless, the major strengths of this study were its prospective study design with standardized patient assessment and stroke classification. Moreover, the current study; to our knowledge, was the first to identify stroke risk factors among the Egyptian pediatric population in a tertiary care referral center in Greater Cairo.

## Conclusions

When dealing with pediatric strokes, physicians should keep in mind a few key differences from adults that can help with diagnosis and treatment: (1) the occurrence of unusual pediatric presentations; diffuse manifestations (only headache, seizure, or absence of obvious clinical symptoms). (2) For pediatric stroke, a more prominent etiological role for non-atherosclerotic arteriopathies, hematological disorders, and coagulopathies was detected, while adults have more atherothrombotic causes. (3) Multiple risk factors coexist in pediatric ischemic stroke. (4) Nontraumatic, spontaneous ICH, IVH, and SAH in childhood are caused by structural lesions in up to 85% of cases. (5) A thrombophilia evaluation is helpful in every case of pediatric stroke. (6) Children who have had a stroke should undergo neurovascular imaging as soon as possible. (7) Vascular imaging with MRA, can help to identify AVMs or cavernomas that may be the cause of cerebral hemorrhage. Conventional catheter-based angiography, though invasive, should be considered when no other explanation for the hemorrhage has been identified. (8) A decision-making process should be carried out case by case by a multi-specialist group, including the neurologist, neuropediatrician, and neuroradiologist.

## Data Availability

The data sets generated and analyzed during the current study are not publicly available due to institutional limitations, yet they are available from the corresponding author on reasonable request.

## Abbreviations

AIS: Acute ischemic stroke
IS: Ischemic stroke
TOAST: Trial of ORG 10172 in Acute Stroke Treatment
NIHSS: National Institutes of Health Stroke Scale
PedNIHSS: Pediatric
TEE: Transesophageal echocardiogram
TTE: Transthoracic echocardiography
LAA: Large-artery atherosclerosis
MTHFR: Methyltetrahydrofolate reductase
COVID-19: Coronavirus disease 2019
RA: Rheumatoid factor
ANA: Antinuclear antibodies
LA: Lupus anticoagulant
ACLs: Anti-cardiolipins
AVM: Arteriovenous malformation
OCSP: Oxfordshire Community Stroke Project
CVST: Cerebral venous sinus thrombosis
TACS: Total anterior circulation syndrome
PACS: Partial anterior circulation syndrome
LACS: Lacunar syndrome
POCS: Posterior circulation syndrome
HLH: Hemophagocytic lymphohistiocytosis
JRA: Juvenile rheumatoid arthritis
ICH: Intracerebral hemorrhage
HS: Hemorrhagic stroke
ACAIS: Anterior circulation ischemic stroke
PCAIS: Posterior circulation ischemic stroke

## References

[1] Donkor ES (2018). Stroke in the century: a snapshot of the burden, epidemiology, and quality of life. Stroke Res Treat.

[2] Mallick AA, Ganesan V, Kirkham FJ, Fallon P, Hedderly T, McShane T, Parker AP, Wassmer E, Wraige E, Amin S, Edwards HB, Tilling K, O'Callaghan FJ (2014). Childhood arterial ischemic stroke incidence, presenting features, and risk factors: a prospective population-based study. Lancet Neurol.

[3] Lynch JK, Hirtz DG, Deveber G, Nelson KB (2002). Report of the National Institute of Neurological Disorders and Stroke Workshop on Perinatal and Childhood Stroke. Pediatrics.

[4] Hamilton W, Huang H, Seiber E, Lo W (2015). Cost and outcome in pediatric ischemic stroke. J Child Neurol.

[5] Wintermark M, Hills NK, DeVeber GA, Barkovich AJ, Bernard TJ, Friedman NR, Mackay MT, Kirton A, Zhu G, Leiva-Salinas C, Hou Q, Fullerton HJ, VIPS Investigators (2017). Clinical and imaging characteristics of arteriopathy subtypes in children with arterial ischemic stroke: results of the VIPS study. AJNR Am J Neuroradiol.

[6] Lanni G, Catalucci A, Conti L, Di Sibio A, Paonessa A, Gallucci M (2011). Pediatric stroke: clinical findings and radiological approach. Stroke Res Treat.

[7] Chadehumbe MA, Khatri P, Khoury JC, Alwell K, Szaflarski JP, Broderick JP, Kissela BM, Kleindorfer DO (2009). Seizures are common in the acute setting of childhood stroke: a population-based study. J Child Neurol.

[8] Hopewell J, Clarke R (2016). Emerging risk factors for stroke: what have we learned from Mendelian randomization studies?. Stroke.

[9] Sträter R, Becker S, von Eckardstein A, Heinecke A, Gutsche S, Junker R, Kurnik K, Schobess R, Nowak-Göttl U (2002). Prospective assessment of risk factors for recurrent stroke during childhood—a 5-year follow-up study. Lancet.

[10] Nowak-Gottl U, Gunther G, Kurnik K, Sträter R, Kirkham F (2003). Arterial ischemic stroke in neonates, infants, and children: an overview of underlying conditions, imaging methods, and treatment modalities. Semin Thromb Hemost.

[11] Fullerton HJ, Wu YW, Sidney S, Johnston C (2007). Risk of recurrent childhood arterial ischemic stroke in a population-based cohort: the importance of cerebrovascular imaging. Pediatrics.

[12] Morrison C, Aravindan S, Rennie A, Liversedge T (2020). Stroke management in children. Pediatr Anesth.

[13] Dlamini N, Kirkham FJ (2009). Stroke and cerebrovascular disorders. Curr Opin Pediatr.

[14] Amarenco P, Bogousslavsky J, Caplan LR, Donnan GA, Hennerici MG (2009). Classification of stroke subtypes. Cerebrovasc Dis.

[15] Low molecular weight heparinoid, ORG 10172 (danaparoid), and outcome after acute ischemic stroke: a randomized controlled trial. The Publications Committee for the Trial of ORG 10172 in Acute Stroke Treatment (TOAST) Investigators. JAMA. 1998; 279(16):1265–72. 9565006

[16] Radu R, Terecoasă E, Băjenaru O, Tiu C (2017). Etiologic classification of ischemic stroke: where do we stand?. Clin Neurol Neurosurg.

[17] Harris S, Sungkar S, Al Rasyid, Kurniawan M, Mesiano T, Hidayat R. TOAST subtypes of ischemic stroke and its risk factors: a hospital-based study at Cipto Mangunkusumo Hospital Indonesia. Stroke Res Treat. 2018;2018: 1–6. 10.1155/2018/9589831. 10.1155/2018/9589831 PMC6252221 30534355

[18] Fullerton HJ, Stence N, Hills NK, Jiang B, Amlie-Lefond C, Bernard TJ, Friedman NR, Ichord R, Mackay MT, Rafay MF, Chabrier S, Steinlin M, Elkind MSV, deVeber GA, Wintermark M, VIPS Investigators (2018). Focal cerebral arteriopathy of childhood: novel severity score and natural history. Stroke.

[19] Wraige E, Hajat C, Jan W, Pohl KR, Wolfe CD, Ganesan V (2003). Ischemic stroke subtypes in children and adults. Dev Med Child Neurol.

[20] Aerden L, Luijckx GJ, Ricci S, Hilton A, Kessels F, Lodder J (2004). Validation of the Oxfordshire Community Stroke Project syndrome diagnosis derived from a standard symptom list in acute stroke. J Neurol Sci.

[21] Hage V, The NIH (2011). Stroke scale: a window into neurological status. Nurs Spectr.

[22] Ichord RN, Bastian R, Abraham L, Askalan R, Benedict S, Bernard TJ, Beslow L, Deveber G, Dowling M, Friedman N, Fullerton H, Jordan L, Kan L, Kirton A, Amlie-Lefond C, Licht D, Lo W, McClure C, Pavlakis S, Smith SE, Tan M, Kasner S, Jawad AF (2011). Interrater reliability of the pediatric NIH stroke scale (PedNIHSS) in a Multicenter Study. Stroke.

[23] DeLaroche AM, Sivaswamy L, Farooqi A, Kannikeswaran N (2016). Pediatric stroke clinical pathway improves the time to diagnosis in an Emergency Department. Pediatr Neurol.

[24] Lehman LL, Khoury JC, Taylor JM, Yeramaneni S, Sucharew H, Alwell K, Moomaw CJ, Peariso K, Flaherty M, Khatri P, Broderick JP, Kissela BM, Kleindorfer DO (2018). Pediatric stroke rates over 17 years: report from a population-based study. J Child Neurol.

[25] Krishnamurthi RV, deVeber G, Feigin VL, Barker-Collo S, Fullerton H, Mackay MT, O'Callahan F, Lindsay MP, Kolk A, Lo W, Shah P, Linds A, Jones K, Parmar P, Taylor S, Norrving B, Mensah GA, Moran AE, Naghavi M, Forouzanfar MH, Nguyen G, Johnson CO, Vos T, Murray CJ, Roth GA, GBD 2013 Stroke Panel Experts Group (2015). Stroke prevalence, mortality and disability-adjusted life years in children and youth aged 0–19 years: data from the global and regional burden of stroke 2013. Neuroepidemiol.

[26] Chiang K, Cheng C (2018). Epidemiology, risk factors and characteristics of pediatric stroke: a nationwide population-based study. QJM.

[27] Donnan GA, Fisher M, Madeod M, Davis SM (2008). Stroke [Seminar]. Lancet.

[28] Feigin V, Lawes C, Bennet D, Barker-Cello S, Parag V (2009). Worldwide stroke incidence and early case fatality in 56 population based studies: a systematic review. Lancet Neurol.

[29] Kleindorfer DO, Towfighi A, Chaturvedi S, Cockroft KM, Gutierrez J, Lombardi-Hill D, Kamel H, Kernan WN, Kittner SJ, Leira EC, Lennon O, Meschia JF, Nguyen TN, Pollak PM, Santangeli P, Sharrief AZ, Smith SC, Turan TN, Williams LS (2021). 2021 Guideline for the Prevention of Stroke in Patients With Stroke and Transient Ischemic Attack A Guideline From the American Heart Association/American Stroke Association. Stroke.

[30] deVeber GA, Kirton A, Booth FA, Yager JY, Wirrell EC, Wood E, Shevell M, Surmava AM, McCusker P, Massicotte MP, MacGregor D, MacDonald EA, Meaney B, Levin S, Lemieux BG, Jardine L, Humphreys P, David M, Chan AK, Buckley DJ, Bjornson BH (2017). Epidemiology and outcomes of arterial ischemic stroke in children: the Canadian Pediatric Ischemic Stroke Registry. Pediatr Neurol.

[31] Tan KS, Navarro JC, Wong KS, Huang YN, Chiu HC, Poungvarin N, Ryu SJ, Bitanga E, Suwanwela N, Alam SM, Yoon BW (2014). Clinical profile, risk factors and aetiology of young ischaemic stroke patients in Asia: a prospective, multicentre, observational, hospital-based study in eight cities. Neurol Asia..

[32] Golomb MR, Fullerton HJ, Nowak-Gottl U, DeVeber G, International Pediatric Stroke Study Group (2009). Male predominance in childhood ischemic stroke: findings from the International Pediatric Stroke Study. Stroke.

[33] Yock-Corrales A, Babl FE, Mosley IT, Mackay MT (2011). Can the FAST and ROSIER adult stroke recognition tools be applied to confirmed childhood arterial ischemic stroke?. BMC Pediatr.

[34] Zimmer JA, Garg BP, Williams LS, Golomb MR (2007). Age-related variation in presenting signs of childhood arterial ischemic stroke. Pediatr Neurol.

[35] Jeong G, Lim BC, Chae H (2015). Pediatric stroke. J Korean Neurosurg Soc.

[36] Tiwari L, Shekhar S, Bansal A, Kumar S (2021). COVID-19 associated arterial ischemic stroke and multisystem inflammatory syndrome in children: a case report. Lancet Child Adolesc Health.

[37] Beslow LA, Fox CK, Kossorotoff M, Zambrano YC, Hernández-Chávez M, Hassanein SM, Byrne S, Lim M, Maduaka N, Zafeiriou D, Dowling MM, Felling RJ, Rafay MF, Lehman L, Bernard TJ, Dlamini N (2021). An infrequent complication of SARS-CoV-2. Ann Neurol.

[38] Abd-Allah F, Ibrahim EM, Zidan O, Mohamed MA, Mohamdy AA, Farrag MA, Aboulfotooh AM, Gomaa HA (2018). Screening of asymptomatic intracranial arterial stenosis among high risk subjects: a pilot study from Egypt. J Vasc Interv Neurol.

[39] Soliman R, Oraby M, Fathy M, Essam A (2018). Risk factors of acute ischemic stroke in patients presented to Beni-Suef University Hospital: prevalence and relation to stroke severity at presentation. Egypt J Neurol Psychiatr Neurosurg.

[40] Khedr E, Tony A, Habeel M, Nasreldein A (2021). Frequency and risk factors of carotid artery disease among ischemic stroke patients in the south Egypt: hospital-based study. Egypt J Neurol Psychiatr Neurosurg.

[41] Huang HW, Guo MH, Lin RJ, Chen YL, Luo Q, Zhang Y, Wong KS (2007). Prevalence and risk factors of middle cerebral artery stenosis in asymptomatic residents in Rongqi County. Guangdong Cerebrovasc Dis.

[42] López-Cancio E, Dorado L, Millán M, Reverté S, Suñol A, Massuet A, Galán A, Alzamora MT, Pera G, Torán P, Dávalos A, Arenillas JF (2012). The Barcelona-Asymptomatic Intracranial Atherosclerosis (AsIA) study: prevalence and risk factors. Atherosclerosis.

[43] El Zayat S, Fawzy E, Zaki MA, Zakaria G, Abdel Ghaffar H, Raouf MA (2020). Comparison between intracranial and extracranial arteries using neuroimaging in acute ischemic stroke and its relation to risk factors. IJMA.

[44] Shaikh NA, Bhatty S, Irfan M, Khatri G, Vaswani AS, Jakhrani N (2010). Frequency, characteristics and risk factors of carotid artery stenosis in ischaemic stroke patients at Civil Hospital Karachi. J Pak Med Assoc.

[45] Hassan KM, Verma A, Prakash S, Chandran V, Kumar S, Banerji A (2013). Prevalence and association of lifestyle factors with extracranial carotid atherosclerosis in non-cardioembolic anterior circulation strokes in adult males less than 50 years: one year cross-sectional study. Ann Indian Acad Neurol.

[46] Saber H, Amiri A, Thrift AG, Stranges S, BavarsadShahripour R, Farzadfard MT, Mokhber N, Behrouz R, Azarpazhooh MR (2017). Epidemiology of intracranial and extracranial large artery stenosis in a population-based study of stroke in the Middle East. Neuroepidemiol.

[47] Wang MY, Mimran R, Mohit A, Lavine SD, Giannotta S (2000). Carotid stenosis in a multiethnic population. J Stroke Cerebrovasc Dis.

[48] Wasay M, Azeemuddin M, Masroor I, Sajjad Z, Ahmed R, Khealani BA, Malik MA, Afridi MB, Kamal A (2009). Frequency and outcome of carotid atheromatous disease in patients with stroke in Pakistan. Stroke.

[49] Williams LS, Garg BP, Cohen M, Fleck JD, Biller J (1997). Subtypes of ischemic stroke in children and young adults. Neurology.

[50] Bigi S, Fischer U, Wehrli E, Mattle HP, Boltshauser E, Bürki S, Jeannet PY, Fluss J, Weber P, Nedeltchev K, El-Koussy M, Steinlin M, Arnold M (2010). Differences in risk-factors, aetiology and outcome between children and young adults with acute ischaemic stroke. Neuropediatrics.

[51] Barnes C, Deveber G (2006). Prothrombotic abnormalities in childhood ischaemic stroke. Thromb Res.

[52] Simonetti BG, Rafay MF, Chung M, Lo WD, Beslow LA, Billinghurst LL (2020). Comparative study of posterior and anterior circulation stroke in childhood: results from the International Pediatric Stroke Study. Neurology.

[53] Beslow LA, Ichord RN, Gindville MC, Kleinman JT, Bastian RA, Smith SE, Licht DJ, Hillis AE, Jordan LC (2014). Frequency of hematoma expansion after spontaneous intracerebral hemorrhage in children. JAMA Neurol.

